# Supplementary material for: Whole cervix imaging of collagen, muscle, and cellularity in term and preterm pregnancy
Source: Nat Commun. 2024 Jul 19;15:5942. doi: 10.1038/s41467-024-48680-9 (PMC11271604; doi:10.1038/s41467-024-48680-9)
Supplement: Supplementary file 1 — Supplementary Information [file 41467_2024_48680_MOESM1_ESM.pdf]

Supplementary Materials for
Whole cervix imaging of collagen, muscle, and cellularity in term and preterm pregnancy

The PDF includes:

Results - Supplementary ... 2
Correlation at different gird size ... 2
Correlation analysis of ex vivo specimen between diffusion tensor imaging and histology ... 4
Correlation analysis of ex vivo specimen between free water imaging and histology ... 7
Methods - Supplementary ... 9
Whole cervix DBSI multi-tensor models ... 9
b table for both in vivo and ex vivo imaging ... 10
Ex vivo imaging of cervical tissue blocks ... 11
Human in vivo MR imaging sequences ... 12

Supplementary Figures

Fig S. 1: Pearson correlation analysis of DBSI versus histology at grid sizes of 2.5mm, 2mm, 1.75mm, and 1.5mm for specimen P1-S1. ... 2
Fig S. 2: Pearson correlation analysis of DBSI versus histology at grid sizes of 2.5mm, 2mm, 1.75mm, and 1.5mm for specimen P1-S2. ... 3
Fig S. 3: Pearson correlation analysis of DBSI versus histology at grid sizes of 2.5mm, 2mm, 1.75mm, and 1.5mm for specimen P2-S1. ... 3
Fig S. 4: Pearson correlation analysis of DBSI versus histology at grid sizes of 2.5mm, 2mm, 1.75mm, and 1.5mm for specimen NP1-S1. ... 4
Fig S. 5: Pearson correlation analysis of DTI versus histology for specimen P1-S1. ... 5
Fig S. 6: Pearson correlation analysis of DTI versus histology for specimen P1-S2. ... 5
Fig S. 7: Pearson correlation analysis of DTI versus histology for specimen P2-S1. ... 6
Fig S. 8: Pearson correlation analysis of DTI versus histology for specimen NP1-S1. ... 6
Fig S. 9: Pearson correlation analysis of FWI versus histology for specimen P1-S1. ... 7
Fig S. 10: Pearson correlation analysis of FWI versus histology for specimen P1-S2. ... 7
Fig S. 11: Pearson correlation analysis of FWI versus histology for specimen P2-S1. ... 8
Fig S. 12: Pearson correlation analysis of FWI versus histology for specimen NP1-S1. ... 8
Fig S. 13: Isotropic tensor models for whole cervix DBSI ... 9
Fig S. 14 Custom cervix specimen holder ... 11
Fig S. 15 Custom Helmholtz pair coil ... 11

Supplementary Tables

Table S. 1 Anisotropic tensor models for whole cervix DBSI ... 9
Table S. 2: *b* table for in vivo and ex vivo imaging ... 10

Results - Supplementary

Correlation at different gird size

The chosen 2.5 × 2.5 mm grid size, as mentioned in the main text, aligns with the standard in vivo diffusion MRI resolutions in clinical MR scanners. To reinforce the validity of our findings in the main text, conducted additional analyses using different grid sizes (2 × 2 mm, 1.75 × 1.75 mm, and 1.5 × 1.5 mm). These analyses (Fig S. 1 – Fig S. 4) consistently demonstrate a strong correlation between histology quantification and ex vivo DBSI metrics, underscoring the robustness of our approach.

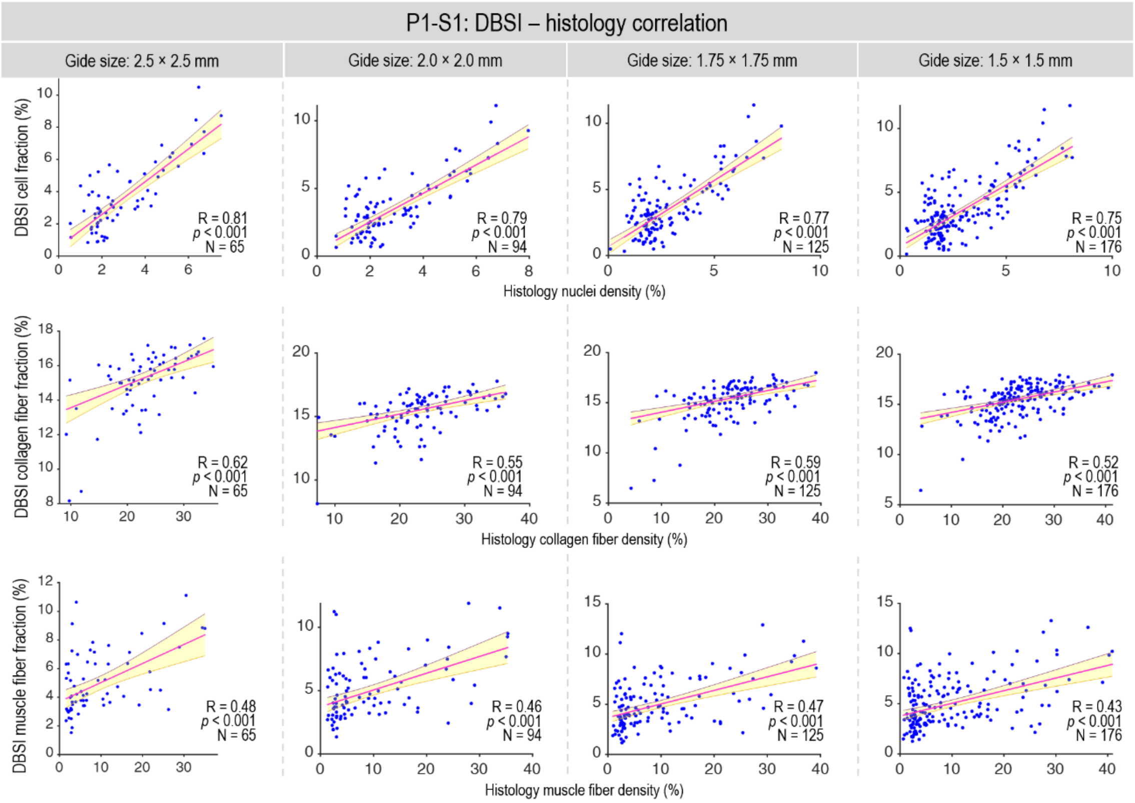

Fig S. 1: Correlation analysis of DBSI versus histology at grid sizes of 2.5mm, 2mm, 1.75mm, and 1.5mm for specimen P1-S1. Data points indicate mean values per grid box from Fig. 2, 3, and 4, panels B and C. Pearson correlation: two-sided, at 0.05 significance level; Red lines: linear fits; shaded areas: 95% confidence intervals.

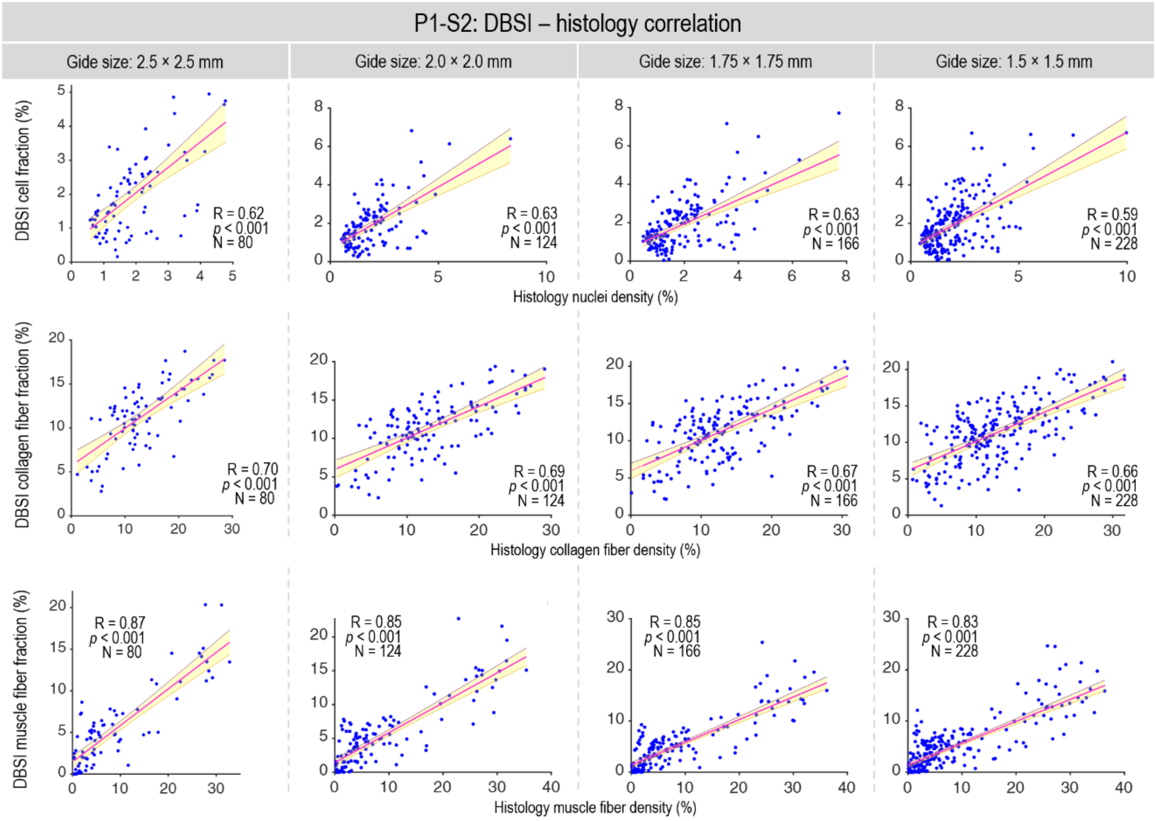

Fig S. 2: Correlation analysis of DBSI versus histology at grid sizes of 2.5mm, 2mm, 1.75mm, and 1.5mm for specimen P1-S2. Data points indicate mean values per grid box from Fig. 2, 3, and 4, panels B and C. Pearson correlation: two-sided, at 0.05 significance level; Red lines: linear fits; shaded areas: 95% confidence intervals.

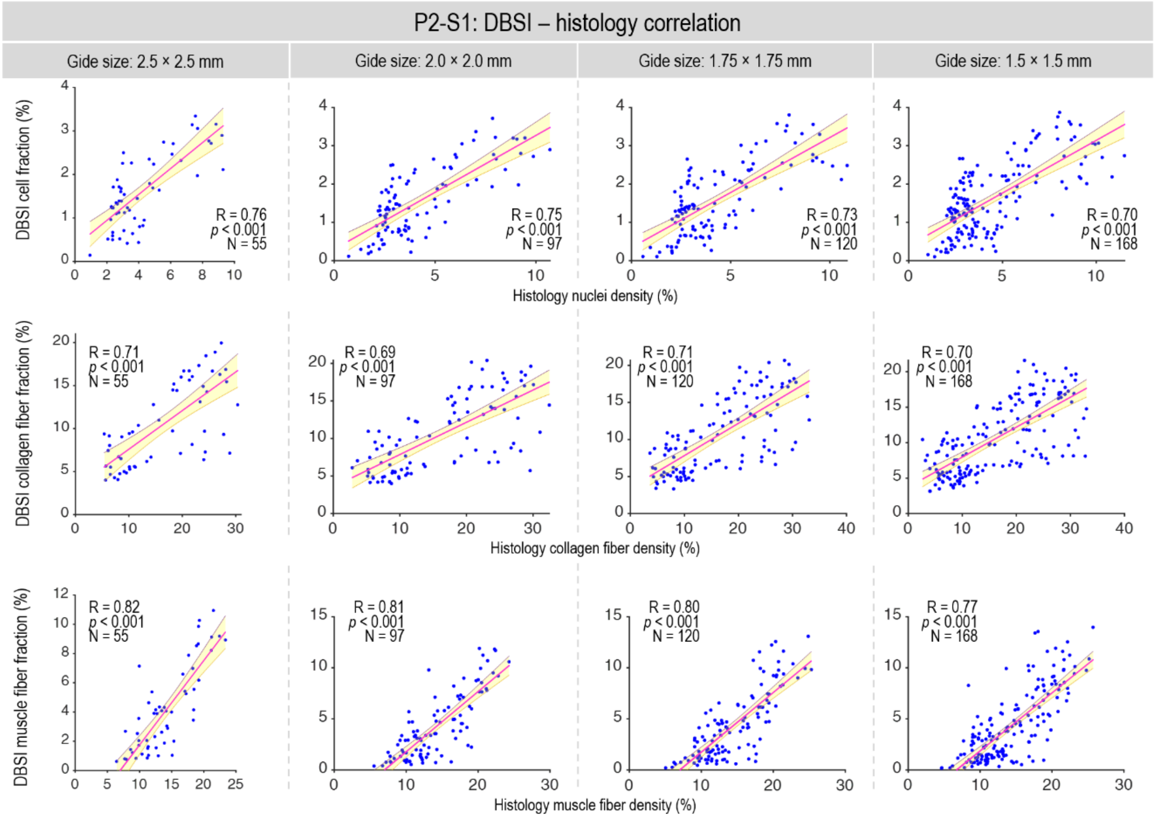

Fig S. 3: Correlation analysis of DBSI versus histology at grid sizes of 2.5mm, 2mm, 1.75mm, and 1.5mm for specimen P2-S1. Data points indicate mean values per grid box from Fig. 2, 3, and 4, panels B and C. Pearson correlation: two-sided, at 0.05 significance level; Red lines: linear fits; shaded areas: 95% confidence intervals.

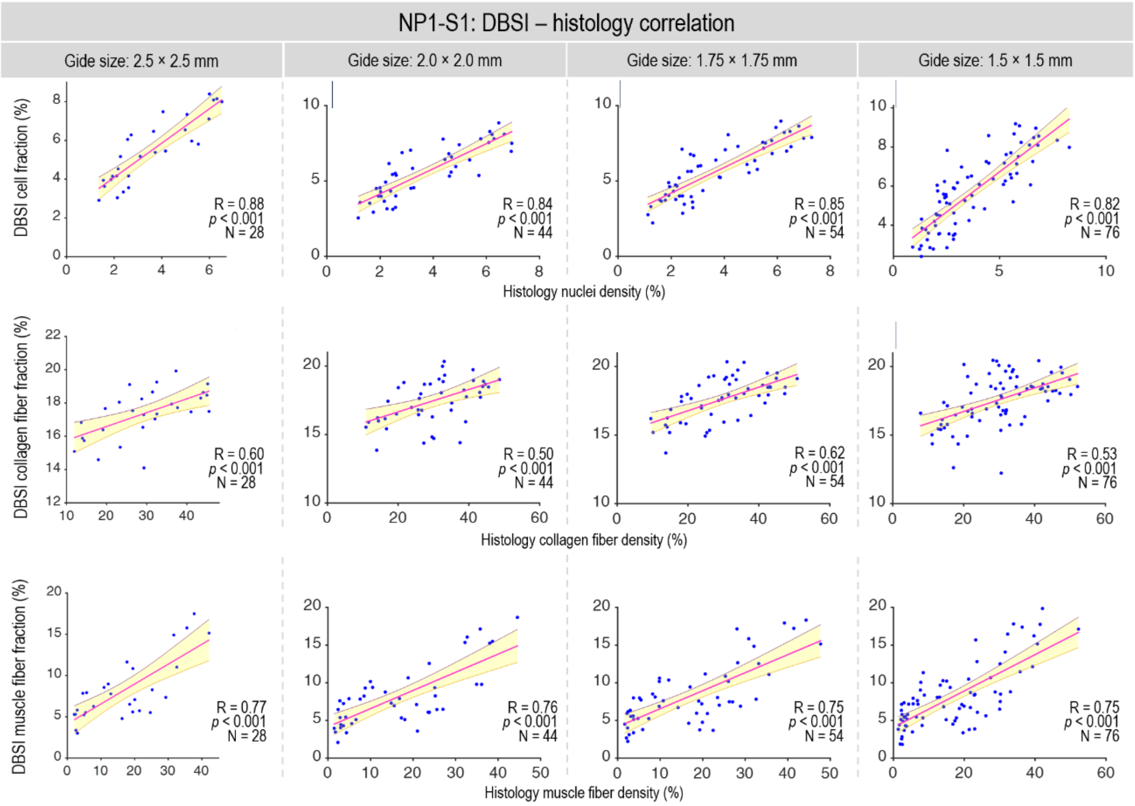

Fig S. 4: Correlation analysis of DBSI versus histology at grid sizes of 2.5mm, 2mm, 1.75mm, and 1.5mm for specimen NP1-S1. Data points indicate mean values per grid box from Fig. 2, 3, and 4, panels B and C. Pearson correlation: two-sided, at 0.05 significance level; Red lines: linear fits; shaded areas: 95% confidence intervals.

Correlation analysis of ex vivo specimen between diffusion tensor imaging and histology

Our supplementary analysis utilizing diffusion tensor imaging (DTI) model (Fig S. 5 – Fig S. 8) reveals that the histology-derived data, including nuclei density indicative of cellularity and collagen fiber density, do not consistently correlate with DTI metrics such as apparent diffusion coefficient (ADC), fractional anisotropy (FA), and axial (AD) and radial diffusivity (RD). Notably, muscle density derived from histology shows a partial correlation with FA. However, given that DTI FA represents the averaged anisotropy from the entire imaging voxel, encompassing both collagen and muscle fibers, it lacks specificity as a biomarker for cervical collagen and muscle fibers.

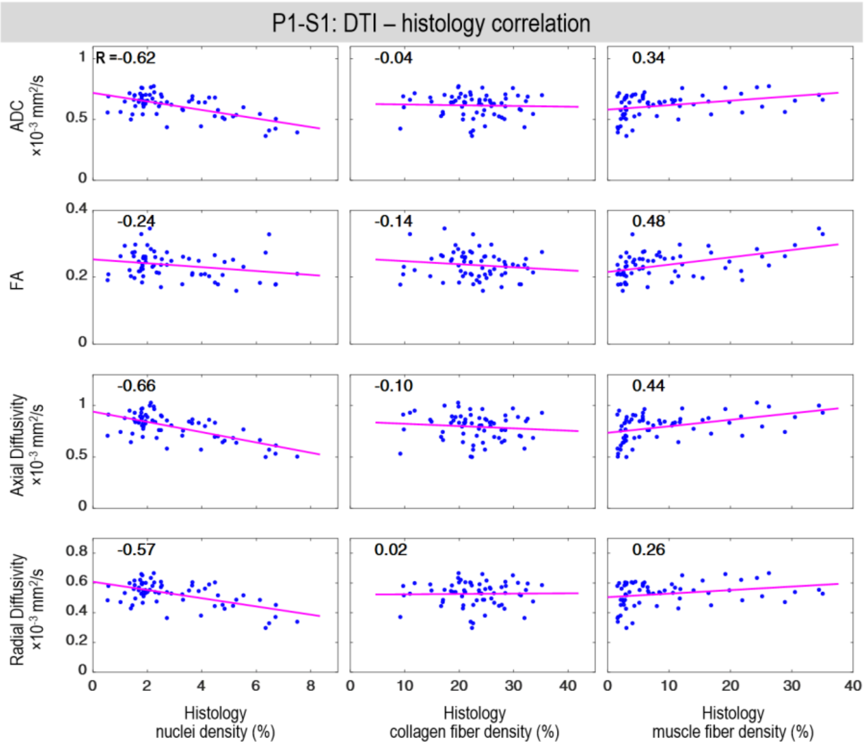

Fig S. 5: Correlation analysis of DTI versus histology for specimen P1-S1. Data points indicate mean values per grid box from Fig. 2, 3, and 4, panels B and C. Pearson correlation: two-sided, at 0.05 significance level; Magenta lines: linear fits.

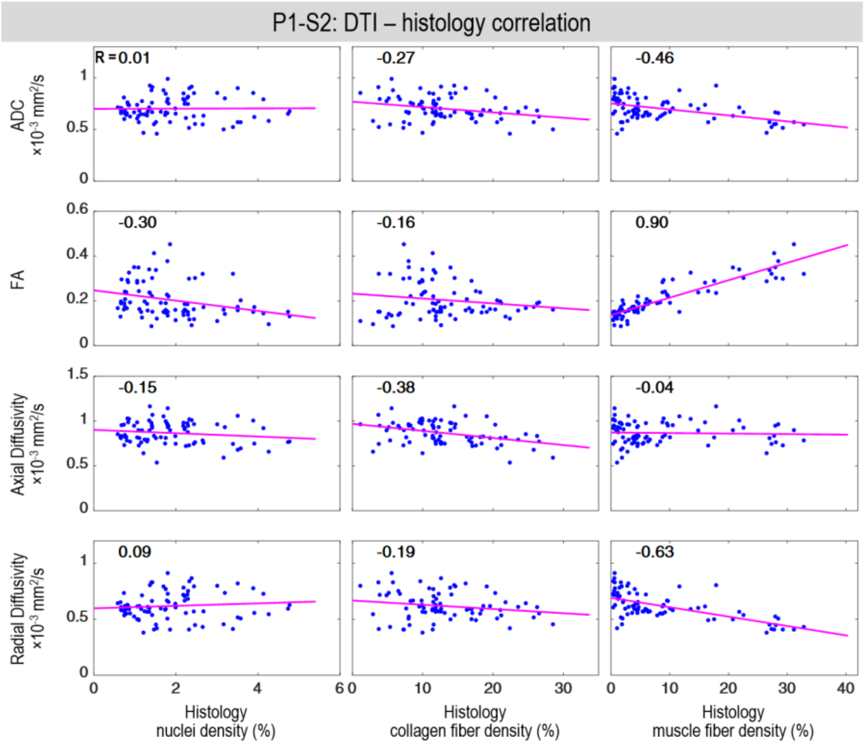

Fig S. 6: Correlation analysis of DTI versus histology for specimen P1-S2. Data points indicate mean values per grid box from Fig. 2, 3, and 4, panels B and C. Pearson correlation: two-sided, at 0.05 significance level; Magenta lines: linear fits.

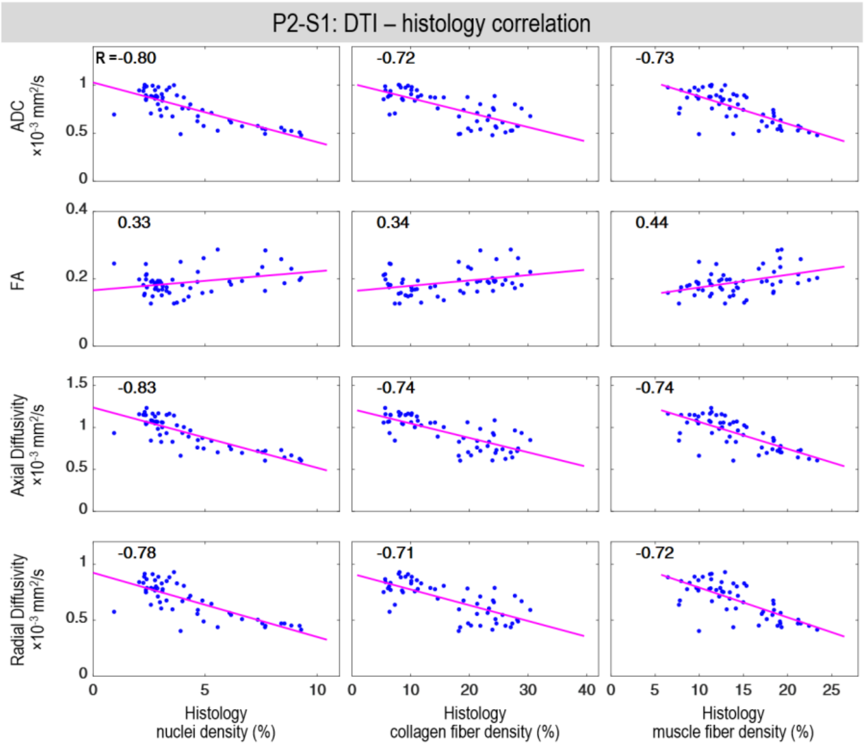

Fig S. 7: Correlation analysis of DTI versus histology for specimen P2-S1. Data points indicate mean values per grid box from Fig. 2, 3, and 4, panels B and C. Pearson correlation: two-sided, at 0.05 significance level; Magenta lines: linear fits.

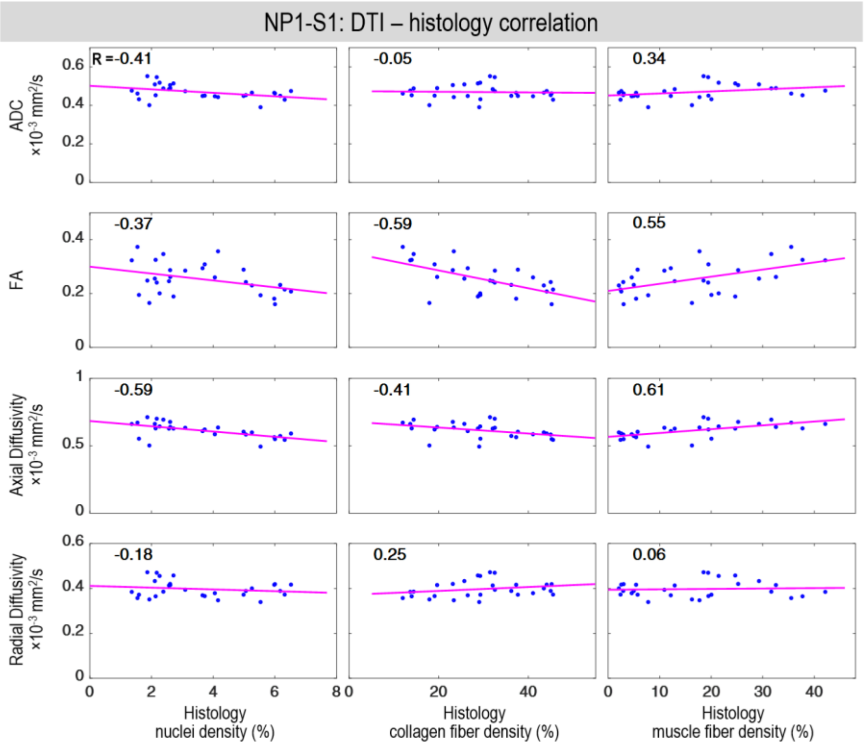

Fig S. 8: Correlation analysis of DTI versus histology for specimen NP1-S1. Data points indicate mean values per grid box from Fig. 2, 3, and 4, panels B and C. Pearson correlation: two-sided, at 0.05 significance level; Magenta lines: linear fits.

Correlation analysis of ex vivo specimen between free water imaging and histology

In the free water imaging (FWI) analysis (Fig S. 9 – Fig S. 12), we observe similar findings as DTI results. The histological data do not show a reliable correlation with FWI metrics.

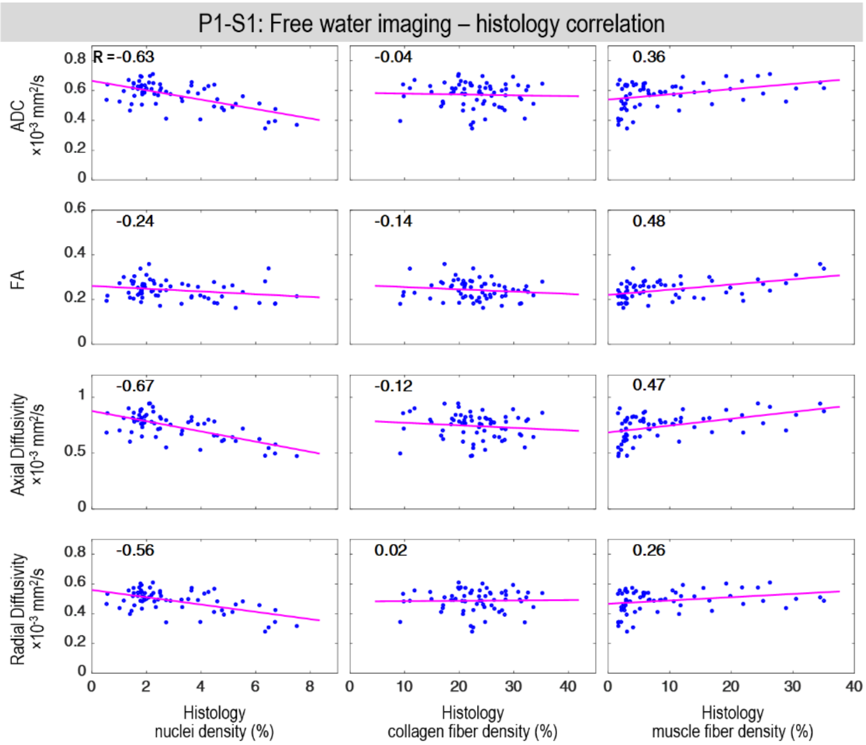

Fig S. 9: Correlation analysis of FWI versus histology for specimen P1-S1. Data points indicate mean values per grid box from Fig. 2, 3, and 4, panels B and C. Pearson correlation: two-sided, at 0.05 significance level; Magenta lines: linear fits.

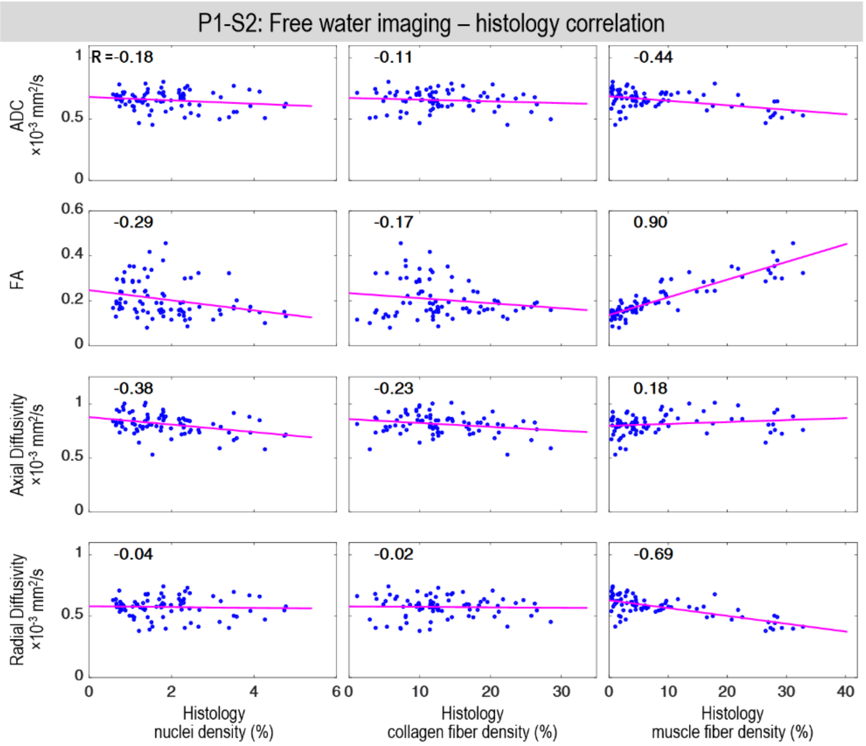

Fig S. 10: Correlation analysis of FWI versus histology for specimen P1-S2. Data points indicate mean values per grid box from Fig. 2, 3, and 4, panels B and C. Pearson correlation: two-sided, at 0.05 significance level; Magenta lines: linear fits.

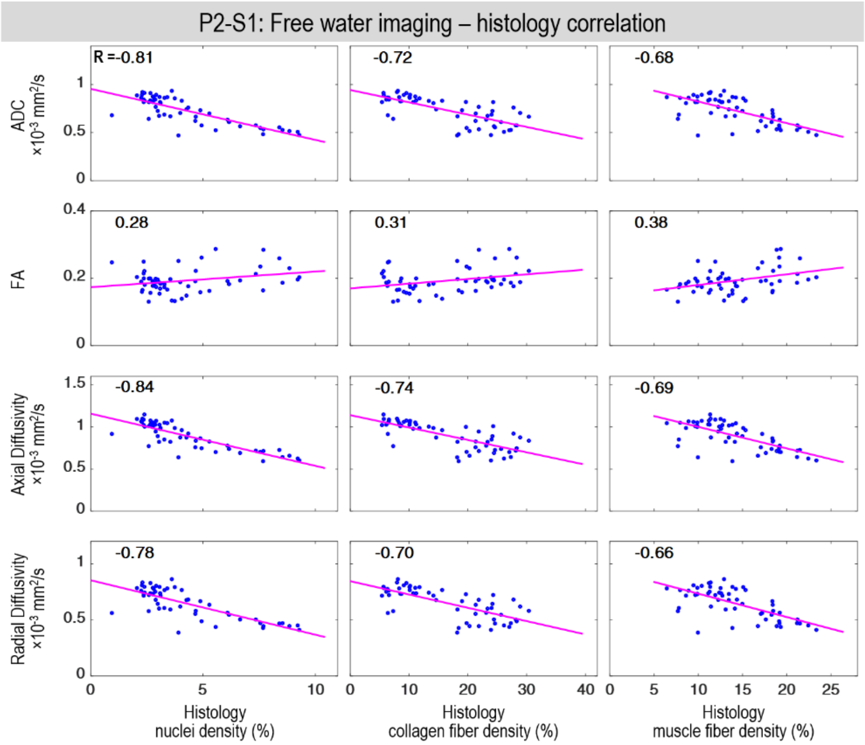

Fig S. 11: Correlation analysis of FWI versus histology for specimen P2-S1. Data points indicate mean values per grid box from Fig. 2, 3, and 4, panels B and C. Pearson correlation: two-sided, at 0.05 significance level; Magenta lines: linear fits.

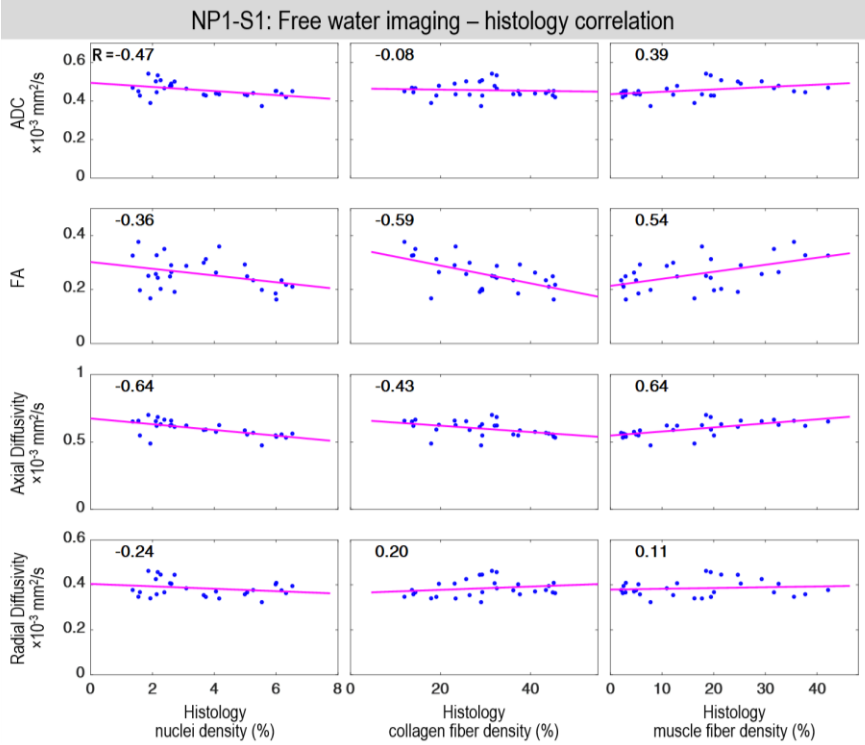

Fig S. 12: Correlation analysis of FWI versus histology for specimen NP1-S1. Data points indicate mean values per grid box from Fig. 2, 3, and 4, panels B and C. Pearson correlation: two-sided, at 0.05 significance level; Magenta lines: linear fits.

Methods - Supplementary

Whole cervix DBSI multi-tensor models

Table S. 1 Anisotropic tensor models for whole cervix DBSI

Diffusivity $\times 10^{-3}$ mm^2/s	Tensor 1	Tensor 2	Tensor 3	Tensor 4	Tensor 5	Tensor 6	Tensor 7	Tensor 8	Tensor 9
Radial Diffusivity (RD)	0.2	0.3	0.4	0.4	0.5	0.6	0.7	0.9	1.2
Axial Diffusivity (AD)	1.2	1.8	2.0	1.6	1.6	1.8	2.1	2.7	3.6
AD / RD	6	6	5	4	3.2	3.0	3.0	3.0	3.0

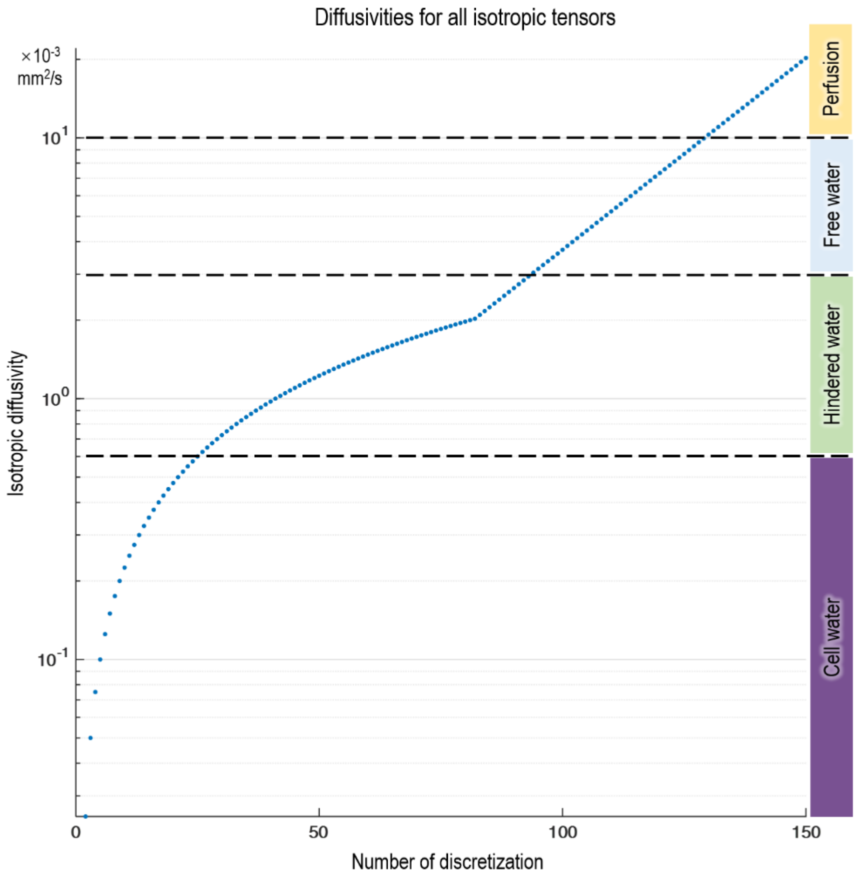

Fig S. 13: Isotropic tensor models for whole cervix DBSI

b table for both in vivo and ex vivo imaging

Table S. 2: *b* table for in vivo and ex vivo imaging

b value in vivo	*b* value ex vivo	*b* vector x	*b* vector y	*b* vector z
0	0	0.000	0.000	0.000
10	20	0.685	0.430	0.589
50	100	0.000	-1.000	0.000
110	250	-0.046	0.895	0.443
200	450	-0.306	0.572	0.761
310	700	0.577	-0.577	0.577
540	1210	0.851	-0.500	0.162
720	1610	-0.526	-0.809	0.262
900	2020	0.000	0.808	-0.589
0	0	0.000	0.000	0.000
1120	2520	0.000	0.809	0.588
1260	2830	0.577	0.577	-0.577
1390	3130	-0.851	0.310	-0.425
1520	3410	-0.851	0.000	0.526
1640	3700	0.526	-0.500	-0.688
1770	3980	0.000	0.000	1.000
1900	4280	0.851	0.000	0.526
1900	4280	0.851	0.000	0.526
2000	4500	0.000	-0.809	-0.588
0	0	0.000	0.000	0.000
20	40	0.887	0.258	0.382
60	140	0.028	-0.693	-0.721
130	300	-0.852	-0.308	0.424
220	500	0.852	0.499	0.161
360	810	0.000	-0.309	-0.951
580	1310	0.000	-0.310	0.951
760	1710	-0.934	-0.357	0.000
940	2120	-0.577	-0.577	-0.577
0	0	0.000	0.000	0.000
1160	2620	0.000	0.934	0.356
1290	2910	0.851	0.500	-0.163
1420	3200	0.000	0.308	0.951
1550	3480	0.357	0.000	0.934
1680	3770	0.851	-0.500	-0.163
1800	4060	0.934	-0.357	0.000
1930	4350	0.851	0.000	-0.526
1800	4060	0.934	-0.357	0.000
1930	4350	0.851	0.000	-0.526
2000	4500	0.000	-0.809	-0.588
0	0	0.000	0.000	0.000
20	50	0.129	0.799	-0.588
80	180	-0.526	-0.236	-0.817
160	360	-0.272	-0.962	-0.039
270	600	0.000	-0.934	-0.358
400	910	0.357	0.000	-0.934
630	1410	-0.851	0.309	0.425
810	1810	-0.526	0.000	0.851
1030	2320	-0.850	0.000	-0.526
0	0	0.000	0.000	0.000
1200	2700	0.000	0.934	-0.357
1320	2980	-0.934	0.357	0.000
1450	3270	0.000	0.310	-0.951
1580	3560	-0.851	-0.309	-0.425
1710	3850	-0.526	-0.809	-0.263
1840	4140	0.526	0.000	-0.851
1970	4420	-0.357	0.000	0.934
1710	3850	-0.526	-0.809	-0.263
1840	4140	0.526	0.000	-0.851
1970	4420	-0.357	0.000	0.934
2000	4500	0.000	-0.809	-0.588
0	0	0.000	0.000	0.000
30	70	0.887	0.261	0.380
90	200	0.577	-0.577	-0.577
180	400	-0.357	0.000	-0.934
290	660	0.147	-0.280	0.949
490	1110	0.000	-0.934	0.356
670	1510	-0.526	0.809	0.262
850	1910	-0.526	0.500	-0.688
1080	2420	0.526	0.501	0.687
0	0	0.000	0.000	0.000
1230	2760	-0.526	0.809	-0.263
1360	3050	0.934	0.357	0.000
1480	3340	-0.526	0.000	-0.851
1610	3630	-0.526	-0.500	0.688
1740	3920	1.000	0.000	0.000
1870	4200	0.526	0.000	0.851
2000	4490	0.000	-0.809	0.588
1610	3630	-0.526	-0.500	0.688
1740	3920	1.000	0.000	0.000
1870	4200	0.526	0.000	0.851
2000	4490	0.000	-0.809	0.588
2000	4500	0.000	-0.809	-0.588

Ex vivo imaging of cervical tissue blocks

The tissue block was embedded in 2% agar gel in the custom specimen holder and then loaded into the custom-made Helmholtz pair coil.

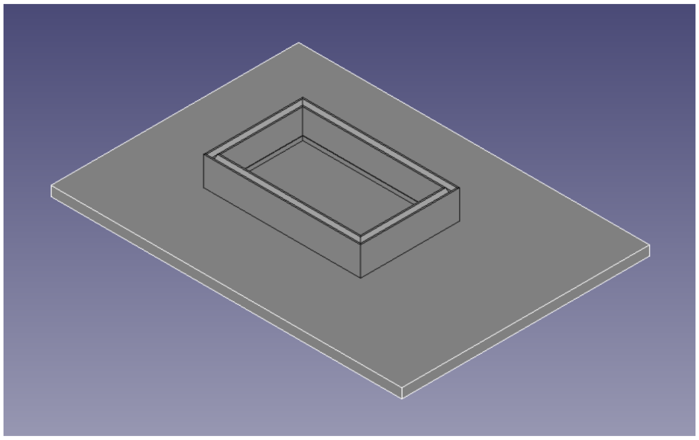

Fig S. 14 Custom cervix specimen holder

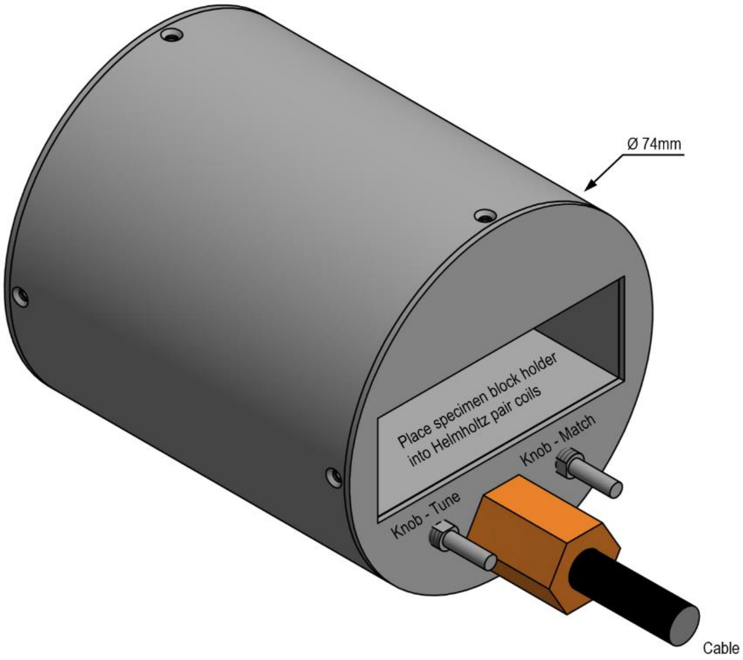

Fig S. 15 Custom Helmholtz pair coil

Human in vivo MR imaging sequences

1) localizer
2) T1-weighted volume-interpolated breathhold examination (VIBE) sequence in the sagittal plane, with the following parameters: repetition time (TR) = 4.07 ms; echo time (TE) = 1.87 ms; field of view (FOV) = 400 × 400 mm; data matrix, 320 × 320; layer thickness, 4.0mm; slice spacing, 0.8 mm; number of layers, 80; flip angle, 10°.
3) T2-weighted turbo spin echo high-resolution images in sagittal plane, with the following parameters: TR = 4660 ms; TE = 96 ms; FOV, 350 × 350 mm; data matrix, 320 × 650; layer thickness, 4.0 mm; slice spacing, 0.8 mm; number of layers, 34; flip angle, 155°.
4) T2-weighted turbo spin echo high-resolution images in oblique planes perpendicular to cervical cannel, with the following parameters: TR = 4110 ms; TE = 96 ms; FOV, 400 × 400 mm; matrix, 320 × 650; layer thickness, 4.0 mm; slice spacing, 0.8 mm; number of layers, 30; flip angle, 155°.
5) Two dimensional single-shot echo planar imaging diffusion-weighted sequence, with the following parameters: TR = 6300 ms; TE = 63 ms; FOV = 350 × 350 mm; data matrix, 128 × 128; slice thickness, 3.0 mm, no slice spacing. The diffusion gradients were applied in up to 74 directions (same as ex vivo, Table S.2) with B values ranging from 0 - 2000 s/mm^2 .
