# Supplementary material for: Whole cervix imaging of collagen, muscle, and cellularity in term and preterm pregnancy
Source: Nat Commun. 2024 Jul 19;15:5942. doi: 10.1038/s41467-024-48680-9 (PMC11271604; doi:10.1038/s41467-024-48680-9)
Supplement: Supplementary file 3 — Reporting Summary [file 41467_2024_48680_MOESM3_ESM.pdf]

nature portfolio

Corresponding author(s): Yong Wang, Qing Wang

Last updated by author(s): Apr 29, 2024

Reporting Summary

Nature Portfolio wishes to improve the reproducibility of the work that we publish. This form provides structure for consistency and transparency in reporting. For further information on Nature Portfolio policies, see our Editorial Policies and the Editorial Policy Checklist.

Statistics

For all statistical analyses, confirm that the following items are present in the figure legend, table legend, main text, or Methods section.

n/a	Confirmed	
☐	☒	The exact sample size (*n*) for each experimental group/condition, given as a discrete number and unit of measurement
☐	☒	A statement on whether measurements were taken from distinct samples or whether the same sample was measured repeatedly
☐	☒	The statistical test(s) used AND whether they are one- or two-sided<br>*Only common tests should be described solely by name; describe more complex techniques in the Methods section.*
☒	☐	A description of all covariates tested
☒	☐	A description of any assumptions or corrections, such as tests of normality and adjustment for multiple comparisons
☐	☒	A full description of the statistical parameters including central tendency (e.g. means) or other basic estimates (e.g. regression coefficient) AND variation (e.g. standard deviation) or associated estimates of uncertainty (e.g. confidence intervals)
☐	☒	For null hypothesis testing, the test statistic (e.g. *F*, *t*, *r*) with confidence intervals, effect sizes, degrees of freedom and *P* value noted<br>*Give P values as exact values whenever suitable.*
☒	☐	For Bayesian analysis, information on the choice of priors and Markov chain Monte Carlo settings
☒	☐	For hierarchical and complex designs, identification of the appropriate level for tests and full reporting of outcomes
☐	☒	Estimates of effect sizes (e.g. Cohen's *d*, Pearson's *r*), indicating how they were calculated

Our web collection on statistics for biologists contains articles on many of the points above.

Software and code

Policy information about availability of computer code

Data collection	Ex vivo MR images were acquired on Varian 11.7T MR scanner with Vnmrj 4.2 software. Histology images were digitized by Hamamatsu NanoZoomer HT. In vivo MR images were acquired on Siemens 3 Tesla MAGNETOM Vida.
Data analysis	Ex vivo image registration and analysis were perform in 3D slicer 5.2.1 and MATLAB 2022b. In vivo image analysis were perform in 3D slicer 5.2.1 and MATLAB 2022b. DBSI analysis were conducted within MATLAB 2022b.

For manuscripts utilizing custom algorithms or software that are central to the research but not yet described in published literature, software must be made available to editors and reviewers. We strongly encourage code deposition in a community repository (e.g. GitHub). See the Nature Portfolio guidelines for submitting code & software for further information.

Data

Policy information about availability of data

All manuscripts must include a data availability statement. This statement should provide the following information, where applicable:

- Accession codes, unique identifiers, or web links for publicly available datasets
- A description of any restrictions on data availability
- For clinical datasets or third party data, please ensure that the statement adheres to our policy

The correlation and statistical data supporting the findings of this study are available within the paper, the Supplementary Materials, and the Source Data file. The DBSI and histology maps in this study are deposited at Figshare: https://doi.org/10.6084/m9.figshare.25584081.

Research involving human participants, their data, or biological material

Policy information about studies with human participants or human data. See also policy information about sex, gender (identity/presentation), and sexual orientation and race, ethnicity and racism.

Reporting on sex and gender	The study is only applicable to females.
Reporting on race, ethnicity, or other socially relevant groupings	The study does not emphasize race and ethnicity comparisons; consequently, in the in vivo study, the term and preterm groups were similar with regard to maternal age and race (summarized in Table 1 in the main text).
Population characteristics	In the ex vivo study, two multiparous patients (who provided samples P1-S1, P1-S2, and P2-S1) were both 37 years of age and were undergoing hysterectomy after delivery. One patient (sample NP1-S1) was 43 years of age, non-pregnant, and undergoing hysterectomy to treat long-term abnormal uterine bleeding.<br>In the in vivo study, population characteristics are summarized in Table 1 in the main text.
Recruitment	The ex vivo specimen collection was approved by the Washington University in St. Louis Human Research Protection Office (HRPO: 201903056, 202006074). Eligible pregnant participants in the ex vivo study included those carrying a single fetus with normal fetal anatomy, diagnosed with placenta accreta, planning to deliver at Barnes-Jewish Hospital (Saint Louis, Missouri), English-speaking, and aged 18 years or older. Pregnant participants were excluded if they had MRI contraindications or if the fetus presented significant anomalies. Non-pregnant participants eligible for the ex vivo study were English-speaking, aged 18 to 45 years, and scheduled for a medically-indicated or prophylactic hysterectomy at Barnes-Jewish Hospital (Saint Louis, Missouri). Exclusion criteria for non-pregnant participants included MRI contraindications, known cervical anomalies, or a diagnosis of gynecological and/or metastatic cancer. All cervix specimens from all enrolled participants provided by Barnes-Jewish Hospital post-hysterectomy were included in the analysis. There are no self-selection processes in our work. Therefore, there are no self-selection bias or other biases impacting our results.<br><br>The parent study from which the in vivo data for this study were obtained was approved by the Washington University in St. Louis Human Research Protection Office (HRPO: 201707152, 202006021). Patients were eligible if they were pregnant with a single fetus with a normal fetal anatomy and intended to deliver at Barnes-Jewish Hospital (Saint Louis, Missouri), were English-speaking, and were aged 18 years or older. Patients were excluded from the parent study if they tested positive for a blood-borne infectious disease, were an intravenous drug user, had contraindications to undergoing an MRI, had a body mass index greater than 40, or if the fetus had significant anomalies.<br><br>In this retrospective in vivo study, the following inclusion criteria were applied: For the preterm cohort with inflammation-associated adverse conditions, participants must have delivered before 37 weeks of gestation. Additionally, they must have had one or more of the following: a positive test result for gonorrhea, chlamydia, trichomoniasis, syphilis, human papillomavirus, bacterial vaginosis, yeast infection, herpes simplex virus, or COVID-19; a positive test for Group B Streptococcus (GBS) during pregnancy or receiving treatment for GBS; or diagnosed with endometriosis, placenta previa/ accreta, or chorioamnionitis. For the healthy term cohort, participants must have delivered at or beyond 37 weeks of gestation and must not have had any of the inflammation-related adverse conditions described for the preterm group.
Ethics oversight	All procedures were approved and performed in accordance with the ethical standards of the Washington University in St. Louis Institutional Review Board (IRB) through the Human Research Protections Office.

Note that full information on the approval of the study protocol must also be provided in the manuscript.

Field-specific reporting

Please select the one below that is the best fit for your research. If you are not sure, read the appropriate sections before making your selection.

☒ Life sciences ☐ Behavioural & social sciences ☐ Ecological, evolutionary & environmental sciences

For a reference copy of the document with all sections, see nature.com/documents/nr-reporting-summary-flat.pdf

Life sciences study design

All studies must disclose on these points even when the disclosure is negative.

Sample size	To validate our method, we compared our imaging results with quantitative histology across 228 independent grid boxes from entire cervix specimens of patients, finding a strong correlation ($p < 0.001$).<br><br>Further analysis with smaller grid sizes increased the number of grid boxes to 648 (Fig S. 1 – Fig S. 4 in the supplementary materials), reinforcing the strong statistical correlation between histology quantification and ex vivo DBSI measures ($p < 0.001$).<br><br>We demonstrated the feasibility of in vivo whole cervix DBSI in ten healthy term patients and seven preterm patients with inflammation-associated conditions. The results showed significant differences between two groups ($p < 0.001$ and $p = 0.033$) and distinct separation in 95% probability ellipsoids of three DBSI measures.
Data exclusions	From the initial cohort of ten preterm patients meeting the inclusion criteria (as detailed in the main text), three were excluded owing to inadequate image quality and severe motion blur. Consequently, seven preterm patients were incorporated into the final analysis.

Replication	The DBSI results are reproducible from the original data.
Randomization	This study aims to develop, validate, and demonstrate the clinical feasibility of a new imaging method, rather than testing a clinical hypothesis through a randomized trial with a large cohort; thus, randomization was not applicable.
Blinding	Given this study is not a clinical trial and involves no clinical intervention, blinding is not applicable in our study design.

Reporting for specific materials, systems and methods

We require information from authors about some types of materials, experimental systems and methods used in many studies. Here, indicate whether each material, system or method listed is relevant to your study. If you are not sure if a list item applies to your research, read the appropriate section before selecting a response.

Materials & experimental systems

n/a	Involved in the study	
☒	☐	Antibodies
☒	☐	Eukaryotic cell lines
☒	☐	Palaeontology and archaeology
☒	☐	Animals and other organisms
☒	☐	Clinical data
☒	☐	Dual use research of concern
☒	☐	Plants

Methods

n/a	Involved in the study	
☒	☐	ChIP-seq
☒	☐	Flow cytometry
☒	☐	MRI-based neuroimaging

Plants

Seed stocks	N/A
Novel plant genotypes	N/A
Authentication	N/A
